# Immune checkpoint inhibitor induced myocarditis, myasthenia gravis, and myositis: A single‐center case series

**DOI:** 10.1002/cam4.5050

**Published:** 2022-09-21

**Authors:** Joshua Longinow, Mohammad Zmaili, Warren Skoza, Nicholas Kondoleon, Robert Marquardt, Cassandra Calabrese, Pauline Funchain, Rohit Moudgil

**Affiliations:** ^1^ Department of Internal Medicine Cleveland Clinic Foundation Cleveland Ohio USA; ^2^ Section of Clinical Cardiology, Department of Cardiovascular Medicine, Heart and Vascular Institute Cleveland Clinic Foundation Cleveland Ohio USA; ^3^ Division of Neuromuscular Center Cleveland Clinic Foundation Cleveland Ohio USA; ^4^ Section of Rheumatologic and Immunologic Disease Cleveland Clinic Foundation Cleveland Ohio USA; ^5^ Taussig Cancer Institute and Case Comprehensive Cancer Center Cleveland Clinic Foundation Cleveland Ohio USA

**Keywords:** immune‐checkpoint inhibitor, immune‐related adverse event, myasthenia gravis, myocarditis, myositis, overlap syndrome

## Abstract

**Background:**

Immune checkpoint inhibitors can result in overlap syndrome comprised of myasthenia gravis, myositis and myocarditis. However, the mortality predictors have not been clearly delineated.

**Methods:**

We examined the characteristics of 11 patients diagnosed with overlap syndrome at Cleveland Clinic. All the available clinical, diagnostic, biochemical and disease specific factors were examined. Clinical predictors of increased mortality were using student *t*‐test for parametric data and Wilcoxon‐signed rank testing for nonparametric data.

**Results:**

Seven patients out of eleven patients were alive during the analysis. Our study did confirm that troponins were indicator of early demise. However, study showed that elevated creatinine, BUN, and decreased hemoglobin were also observed in patients who met early demise. Unlike previously published studies, elevated NT Pro‐BNP and reduced left ventricular ejection fraction were not a seen in this study. However, there were higher incidence of electrical abnormalities in deceased patients when compared to alive.

**Conclusion:**

Our study is first to examine various clinical parameters of overlap syndrome that might be predictive of mortality. This study confirms troponin as possible predictor and adds elevated creatinine, BUN and reduced hemoglobin as possible early biomarkers in deceased patients. The analysis showed that reduced LVEF was not a seen in deceased patients.

## Graphical abstract

Inclusion criteria.

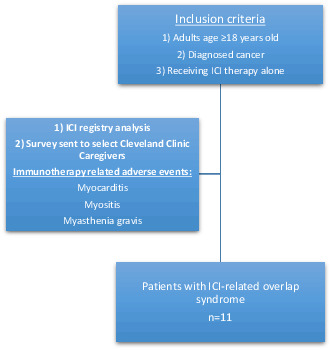

## 1 INTRODUCTION

Immune checkpoint inhibitor therapy (ICI) represents a novel and rapidly changing field in the treatment of cancer. In simplistic terms, immune‐therapy are targeted therapies that act on the immune system through a variety of mechanisms to create a more robust host immune response against cancer cells. Immune checkpoint‐inhibitors are a distinct and novel form of immune‐therapy, targeting the proteins programmed death cell protein 1 (PD‐1), programmed death ligand‐1 (PDL‐1) and cytotoxic T‐lymphocyte‐4 (CTLA‐4), leading to activation of cytotoxic T‐lymphocytes against cancer cells.^1^, ^2^, ^3^ As the range and use of these ICI therapies expands to more cancers, there has been increasing recognition of ICI‐related adverse events (irAE's). These adverse events can occur in any organ system and often with multiple organ toxicities occurring simultaneously. Prior meta‐analyses have noted the frequent co‐occurrence of myasthenia gravis (up to 10%) with myocarditis, and myositis with myocarditis.^4^, ^5^ More recently, cases reports and case series have emerged describing an ICI‐related ‘overlap syndrome’ of myocarditis, myasthenia gravis, and myositis.^6^, ^7^, ^8^, ^9^, ^10^, ^11^

Rates of ICI myocarditis have increasingly been reported and development of ICI‐myocarditis alone is associated with high rate of (upwards of 50%) mortality, with more severe ICI‐myocarditis associated with the combined use of anti‐PD1 and CTLA‐4 therapies.^12^, ^13^ Given its high mortality rate, prognostication becomes even more important in these cases. Prior studies have demonstrated that biomarkers, specifically AST, creatinine, as well as LVEF are predictive of the development of cardiac dysfunction following cancer chemotherapy.^14^ In ICI‐related myocarditis, similar to other cardiac disease entities, troponin‐T levels are of prognostic value, and higher levels (≥1.5) have been associated with increased risk of MACE.^15^ ICI‐related overlap syndrome, however, represents a unique clinical entity from ICI‐related myocarditis or chemotherapy‐related cardiac dysfunction.

In those patients who develop immune‐related overlap syndrome outcomes tend to poor overall.^6^, ^7^, ^8^, ^9^, ^10^, ^11^ With increased use and applications for ICI‐therapies and continued reports of ICI overlap syndrome, it is increasingly important to identify clinical features and patient factors which may portend to a poor prognosis in patients who develop this unique syndrome. The goal of this study was several‐fold. We aimed to evaluate a series of patients with ICI‐related overlap syndrome in order to (1) describe patient characteristics and clinical features including laboratory and biomarker data, and (2) describe overall clinical outcomes, in an effort to elucidate trends that might be of utility in prognostication in patients who develop this ICI‐overlap phenomenon. This case series, to our knowledge, is the largest number of patients published yet.

## 2 METHODS

This study was a single‐center case series review of patients with ICI‐related myocarditis, myositis, myasthenia gravis overlap syndrome. As per institutional guidelines, IRB approval is not required for case reports and/or case series. Furthermore, case series are exempted from consent requirements as per IRB. The study conforms to the standards set by the Declaration of Helsinki and US Federal policy for the protection of human subjects

### 2.1 Study population

Patients were selected from the ICI‐registry database at Cleveland Clinic. Patients were included for study review if they were (1) ≥ 18 years old, (2) had received ICI therapy, (3) carried an ICD‐10 diagnosis of either myocarditis, myositis, and myasthenia gravis overlap syndrome. Chart review and abstraction of patient data for those patients meeting the above criteria was then conducted from our ICI registry and by review of our institutions electronic medical record (EMR). Individual patients were identified as having ICI overlap syndrome by review for associated ICD‐10 diagnosis codes for myositis, myasthenia gravis, and myocarditis. Following review, a total of 11 patients were identified as meeting criteria for inclusion.

### 2.2 Patient characteristics and outcomes

Patient data obtained included baseline demographic data, immune checkpoint inhibitor received, number of doses received prior to onset of irAE (defined as time to onset of symptoms and/or hospitalization from first dose of irAE), and specific therapies given for irAE overlap syndrome (Table 1 and Table 2). Laboratory, echocardiographic, angiographic, and cardiac MRI (CMRI) data were obtained and reviewed from the time of first presentation with overlap syndrome and/or time of first hospitalization (Table 2 and Table 3). Diagnosis of immune‐related myocarditis was confirmed by EMR review of patient clinical features. We followed the criteria for the diagnosis of immune‐related myocarditis proposed by Bonaca et. al, and patients were classified as having either definite, probable, or possible myocarditis, accordingly^1^, ^15^, ^16^, ^17^ (Table S1). EKG, echocardiogram and CMRI reports were all reviewed for changes suggestive of myocarditis (Table 3). EKG's were compared to the most recent EKG that was collected prior to hospitalization, in order to assess for significant changes (i.e. evidence of ST‐deviation, atrioventricular [AV] block, T wave abnormalities and new arrhythmias). Echocardiographic data was assessed for evidence of new regional wall motion abnormalities, new left ventricular dysfunction/reduced ejection fraction, increased wall thickness suggestive of myocardial edema, or new pericardial effusion. Depressed LVEF was defined as <52% by echocardiography. CMRI reports were reviewed for changes suggestive of myocarditis. Myocarditis on CMRI was defined using the proposed Lake Louise criteria for myocarditis.^18^

**TABLE 1:** Baseline demographics and laboratory data at time of initial presentation/hospitalization

	Alive (*n* = 7)	Deceased (*n* = 4)	*p* value
Demographics			
Age, year (mean)	72.8	77	
Ethnicity			
Caucasian	5	4	
African American	1	0	
Sex			
Male	4	3	
BMI (mean)	28.36 ± 2.44	33.69 ± 4.17	0.223
Laboratory data (mean values)			
Hemoglobin	13.7 ± 0.58	11.6 ± 1.01	0.0001
Hematocrit	41.7 ± 1.37	35.6 ± 2.54	
WBC	7.7 ± 0.84	7.0 ± 0.97	
Platelets	267 ± 23.5	295 ± 46.7	
Sodium	139.1 ± 1.3	135 ± 2.4	
BUN	22.6 ± 4.9	31 ± 2.7	<0.004
Creatinine	0.87 ± 0.06	1.29 ± 0.17	<0.001
Creatine Kinase	4095.6 ± 941.5	2608.5 ± 1346.6	0.0048
AST	253.9 ± 38.3	241.8 ± 64.6	
ALT	199.3 ± 22.1	158.3 ± 44.7	<0.0001
ALP	72.3 ± 6.2	131.8 ± 45.4	<0.001
Total bilirubin	0.371 ± 0.06	0.4 ± 0.11	
INR	1 ± 0.36	1.05 ± 0.03	
NT pro BNP	392.3 ± 193.9	5674 ± 3828.6	
Troponin T	1.0248 ± 0.202	3.4175 ± 0.988	0.0023
Aldolase	17.97 ± 5.1	16.75 ± 0	

**TABLE 2:** Clinical characteristics and patient outcomes

	Alive 63%	Deceased 36%	*p*‐value
Immunotherapy agent received			
Pembrolizumab	18%	27%	
Nivolumab	27%	9%	
Darvolumab	18%	none	
Indication for ICI			
	Maxillary sinus SCC	Malignant Melanoma	
	Merckel cell carcinoma	Cutaneous SCC	
	Renal cell carcinoma	Prostate adenocarcinoma	
	Malignant melanoma		
	Lung adenocarcinoma		
	Malignant melanoma		
Concurrent irAEs			
	Dermatitis Sicca	Dermatitis Hepatitis	
LVEF % (mean)			
	62.4 ± 1.9	54.2 ± 6.1	
ACH‐r seropositivity	36%	36%	
Patient outcomes			
Median doses of ICI prior to presentation	1.6 ± 0.5	1.5 ± 0.6	
Hospitalized	55%	36%	
Time to hospitalization after ICI initiation (days)	47.3 ± 24.9	16.7 ± 11.6	*p* = 0.032
Duration of hospitalization (days)	8.1 ± 4.9	17.5 ± 13.0	
Time to death (days)	N/A	77.3 ± 65.9	
Treatment strategies			
Steroids	7	4	
IVIG	4	1	
Plasmapheresis	0	2	
Abatacept	0	1	
Pyridostigmine	4	3	

Abbreviations: ACH‐r, acetylcholine‐receptor; ICI, immune checkpoint inhibitor; irAE, immune‐therapy related adverse event; IVIG, Intravenous immunoglobulin; LVEF, left ventricular ejection fraction.

**TABLE 3:** Diagnosis of ICI‐myocarditis

Patients	Diagnosis (myocarditis)	Pathology	Diagnostic CMRI	Syndrome	Positive biomarkers (troponin T or I, CK MB)	EKG changes	Echo findings suggestive of myocarditis	(−) angiography	PET evidence	Suggestive CMRI
Deceased										
1^a^	Probable	N/A	N/A	Yes	Yes	Yes	Yes	N/A	N/A	N/A
2^b^	Possible	N/A	N/A	Yes	Yes	Yes	No	N/A	N/A	N/A
3^c^	Possible	N/A	N/A	Yes	Yes	Yes	No	N/A	N/A	N/A
4^d^	Possible	N/A	N/A	No	Yes	Yes	No	No	N/A	N/A
Alive										
1^**e**^	Probable	N/A	N/A	Yes	Yes	No	No	N/A	N/A	Yes
2^f^	Possible	N/A	No	Yes	Yes	No	N/A	N/A	N/A	No
3^g^	Possible	N/A	No	Yes	Yes	Yes	No	N/A	N/A	No
4	Possible	N/A	No	Yes	Yes	No	No	N/A	N/A	No
5	Possible	N/A	No	Yes	Yes	No	Yes	N/A	N/A	No
6	Possible	N/A	No	Yes	Yes	No	No	Yes	N/A	N/A
7	Possible	N/A	N/A	Yes	Yes	No	No	N/A	N/A	N/A

Abbreviations: AV, atrioventricular; CMRI, cardiac magnetic resonance imaging; ECV, extracellular volume; LVEF, left ventricular ejection fraction; PET, positron emission tomography; RBBB, right bundle branch block.

^a^ Newly reduced LVEF (36%), global hypokinesis. EKG with new atrial fibrillation.

^b^ EKG with new second‐degree AV block.

^c^ EKG with new third‐degree AV block.

^d^ EKG with new ST elevation in leads I and aVL, reciprocal depressions in II, III, aVF. Coronary angiography with severe stenosis of a small posterolateral branch.

^e^ CMRI with subendocardial enhancement in the mid‐anterior wall on delayed enhancement imaging suggestive of focal myocarditis, additionally, with increased ECV suggesting diffuse myocardial edema.

^f^ EKG with new incomplete RBBB.

^g^ Echo with presumed new small pericardial effusion.

Patients were stratified as being either deceased or living based upon confirmed death date which was gathered by abstraction of patient data through EMR review. Number of hospitalizations for irAE's were assessed within the first year following initiation of ICI therapy. Time to hospitalization was defined as time from first dose of ICI to first hospitalization for irAE. Time to death was assessed from first dose of immune checkpoint inhibitor to death date as documented in the EMR, likewise, cause of death was abstracted from available data in the EMR.

### 2.3 Statistical analyses

Mean values were compared using Student *t*‐test for parametric data and Wilcoxon‐signed rank testing for nonparametric data. Statistical analysis and computation was performed using JMP 16.1 software and SPSS version 27 (Armonk). Figures were constructed on GraphPad Prism 9.

## 3 RESULTS

### 3.1 Patient population

We identified a total of 11 patients who developed ICI overlap syndrome. Baseline demographic data were similar between patient groups (alive versus deceased) with most patients being male (63%) and of Caucasian ethnicity (82%). There were a wide array of underlying cancers noted in our study population (Table 1). Patients received either single agent durvalumab, pembrolizumab or nivolumab. None of the patients in this case series received combination ICI therapy.

### 3.2 Baseline laboratory data

**FIGURE 1:**
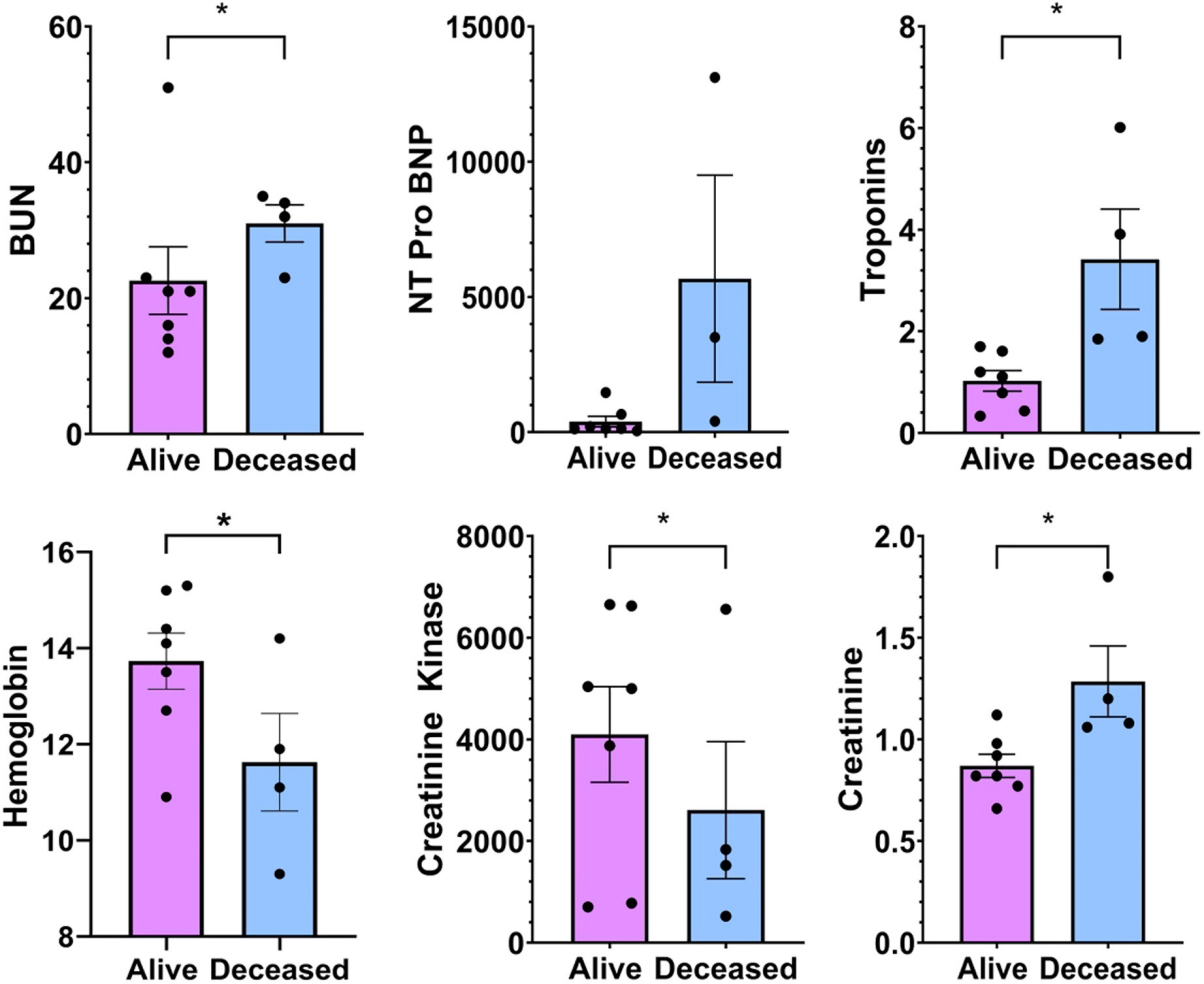
Comparison of relevant biomarkers. Relevant biomarkers assessed at time of presentation and/or hospitalization for irAE. Abbreviations: BUN, blood urea nitrogen; NT pro BNP, N‐terminal pro brain‐natriuretic peptide. ^*^ *p* < 0.05 = significant difference as assessed by Wilcoxon‐test. Values reported as ± SEM.

At time of initial presentation and/or hospitalization, patients who experienced a poor outcome (death) related to overlap syndrome had significantly elevated BUN and creatinine. Creatine kinase (4095.6 ± 941.5 vs. 2608.5 ± 1346.6) and troponin‐T (3.4175 ± 0.988 vs. 1.0248 ± 0.202) were also significantly higher in the deceased group (Table 1 and Figure 1). Deceased patients had significantly lower hemoglobin levels comparatively (Table 1 and Figure 1). We observed trends towards higher NT Pro BNP levels in the deceased group, however this elevation was not statistically significant compared to the alive group (Table 1 and Figure 1).

### 3.3 EKG, Echo and CMRI data

New arrhythmias and/or conduction disturbances were noted more often in the deceased subgroup of patients (36% vs. 18%) (Table 3). Notable changes observed in the deceased group included new‐onset atrial fibrillation with rapid ventricular response, new onset third‐degree AV block, and new ST‐elevation in leads I and aVL. Most patients had normal echocardiography, apart from only one patient in the deceased group who developed new onset LV dysfunction with reduced LVEF to 36%, and global LV hypokinesis. Most patients had normal findings on CMRI, with only one patient who had evidence on delayed enhancement imaging of subendocardial enhancement in the mid‐anterior wall suggestive of focal, atypical myocarditis with mildly increased extracellular volume suggestive of diffuse myocardial edema on CMRI. None of the patients in our study underwent endomyocardial biopsy.

### 3.4 Patient outcomes

The majority (63%) of patients were still alive at the time of this review. Of those who were deceased, 50% were documented to have succumbed to hypoxic respiratory failure and the other patients did not have a documented or identifiable cause for death. The average number of ICI doses prior to the development of overlap syndrome was less than 2, and was similar for both alive and deceased groups, respectively (1.6 vs. 1.5) doses, (Table 2). The overwhelming majority of patients were hospitalized following development of overlap syndrome (90%) (Table 2). Compared to those still alive, deceased patients tended to be hospitalized earlier in relation to the first or second dose of ICI (16 days for deceased versus 43 days for alive), however, the duration of hospitalization was not significantly different between the two groups (Table 2).

## 4 DISCUSSION

ICI‐related overlap syndrome is a unique clinical entity consisting of three co‐existing ICI‐related toxicities, myocarditis, myasthenia gravis and myositis. Case reports and case series describing this ICI overlap phenomenon have emerged in recent years showing that prognosis following the development of this syndrome is poor. Our study represents one of the largest single‐center case‐series of patients with ICI overlap syndrome to date, describing patient characteristics, clinical features, and outcomes.

Overall, 4 out of 11 patients (36%) in this case series died following the development of ICI‐overlap syndrome. Though there was no significant difference in the number of doses of ICI therapy, patients who developed overlap syndrome tended to develop it early (median number of doses prior to onset of overlap syndrome was less than 2). We observed important differences in time to hospitalization following development of ICI‐overlap syndrome. Patients who died were hospitalized significantly earlier compared to those still alive (16 days versus 47 days, *p* = 0.032), following their first or second dose of ICI therapy (Table 2). This trend towards earlier hospitalization for overlap syndrome might suggest a more rapid onset and/or progression of overlap syndrome in patients who experience worse outcomes. Though ICI toxicity may occur at any time during therapy, our findings are in line with prior studies showing that fatal ICI toxicities tend to occur earlier and may be seen within 40 days of initiation of therapy or, shortly after the initial dose.^5^, ^13^

We observed significant differences in biomarker and laboratory data at the time of hospital admission in those patients who died following development of overlap syndrome, as mentioned previously (Table 1). Troponin T was significantly elevated compared to alive patients (3.4175 ± 0.988 vs. 1.0248 ± 0.202, *p* = 0.0023), possibly suggesting that patients who died had a more severe degree of myocarditis. As mentioned previously, prior studies have shown an association between troponin‐T greater than or equal to 1.5 with poor outcomes in patients who developed ICI‐myocarditis. Overall, the aberrations in laboratory data that we observed in patients who had poor outcomes in this case series might suggest that these patients had a greater severity of disease. However, further prospective studies in patients who develop ICI overlap syndrome would be necessary to confirm this, and particularly identify the associations with particular biomarker aberrations with poor outcomes in this unique population.

Most patients in our study could be defined as having “possible myocarditis” as per the criteria proposed by Bonaca et al (Table S1). All patients presented with a syndrome suggestive of myocarditis with either symptoms of chest pain, dyspnea, and/or fatigue and had significant elevations in cardiac biomarkers, and or EKG evidence suggestive of myocarditis. Two patients met proposed criteria for “probable myocarditis” and had findings on echocardiography or CMRI suggesting myocarditis. Most patients in our study had normal echocardiogram findings. Though echocardiography is useful for detection of changes in LV function and wall motion, echocardiogram findings are often normal, particularly in the setting of acute myocarditis thus making it of limited diagnostic value.^17^, ^19^, ^20^ Most patients in our study also had preserved LVEF, and it is worthwhile mentioning that prior studies of patients with ICI myocarditis have shown that increased mortality, though associated with elevated troponin‐T levels, was not linked to reduced LVEF or elevations in NT pro BNP.^15^ Overall, the cardiac findings in our study might suggest that these patients tended to have less severe, or subclinical myocarditis.

Though theories have been postulated regarding the mechanisms underlying the development of irAE's, their biologic basis remains poorly understood and thus, models for the prediction of development of iRAE's based on patient characteristics or tumor features are not well established. In general, it is thought that irAE's may occur when there is (1) antigen cross reactivity between tumor and healthy tissue, (2) release of tumor and healthy tissue antigens following tumor destruction, or (3) direct toxicity from ICI's.^21^ In the case of ICI myocarditis, the mechanism is not fully understood. Myocardial toxicity might occur directly, related to cardiomyocyte expression of PD‐1 and PD‐L1.^16^ Prior studies in mice have shown that knockout of PD‐1 expression was associated with development of autoantibodies towards cardiac troponin‐I and development of autoimmune dilated cardiomyopathy.^22^ Additionally, toxicity might be T‐cell mediated, with particularly high levels of activation of CD8 T‐cells, due to shared T cell antigens between tumor and cardiac myocytes.^20^, ^23^ Indeed, histopathology of endomyocardial samples taken from patients with ICI‐myocarditis generally shows an inflammatory infiltrate consisting predominantly of T‐lymphocytes, similar to findings in non‐ICI related myocarditis and cardiac allograft rejection.^1^ The underlying pathobiology leading to the development of ICI overlap syndrome has not been described to date. Although ICI overlap syndrome is unique from ICI‐myocarditis, it is possible that a similar process involving shared tumor antigens and/or autoantibodies towards cardiac/skeletal muscle, and the neuromuscular junction could lead to the development of this syndrome. Further studies at the biological level will be necessary to elucidate whether such a mechanism exists in the case of ICI‐overlap syndrome.

### 4.1 Limitations

Several limitations should be noted in order to allow for full appreciation of the findings in this study. Firstly, this was a single‐center, retrospective case series review at a large quaternary referral center, which limits the generalizability of this study, and makes this study more liable to introduction of referral bias including referral of a sicker, more advanced patient population. Secondly, selection bias is inherent in the study design (case series) and should be noted. Thirdly, the abstraction of patient data by EMR review and limitations and potential for shortcomings thereof should be noted. Finally, it should be noted that none the patients in our case series had received combination ICI therapy, which is important to mention as combination ICI therapy has been linked to higher mortality, particularly with ICI‐myocarditis as previously described above.^13^

## 5 CONCLUSION

Patients who developed overlap syndrome presented early, after a median of 1.5–1.6 doses of therapy and 36% of the patients in this study died from complications related to overlap syndrome. ICI‐myocarditis in our population tended to be less severe overall. Deceased patients were hospitalized significantly earlier and had significantly higher levels of troponin T, CK, BUN and Cr, and lower hemoglobin levels at the time of hospitalization. These trends are important as they may provide markers for prognostication for future patients who develop this rare condition, though future studies, including prospective studies will be necessary to confirm associations between biomarker alterations and mortality in this syndrome.

## AUTHOR CONTRIBUTIONS

Joshua Longinow: Conceptualization, Visualization, Investigation, writing original draft. Mohammed Zmaili: Methodology, Validation, Reviewing and Editing. Warren Skoza: Validation, Reviewing and Editing. Nicholas Kondoleon: Validation, Reviewing and Editing. Robert Marquardt: Validation (Specifically for Myasthenia Gravis), Reviewing and Editing. Cassandra Calabrese: Validation (Specifically for Myositis), Reviewing and Editing. Pauline Funchain: Validation (Specifically for Oncology), Reviewing and Editing. Rohit Moudgil: Conceptualization, data curation, formal analysis, methodology, project administration, resources, supervision, validation, visualization, writing ‐ original draft, and writing ‐ review and editing.

## FUNDING INFORMATION

No funding was secured for the study.

## CONFLICT OF INTEREST

No Conflict of Interest to disclose for all the authors.

## Supporting information


Table S1

## Data Availability

The data that support the findings of this study are available from the corresponding author upon reasonable request.

## References

[1] Zhang L , Reynolds KL , Lyon AR , Palaskas N , Neilan TG . The evolving immunotherapy landscape and the epidemiology, diagnosis, and management of cardiotoxicity. JACC CardioOncol. 2021;3(1):35‐47. doi:10.1016/j.jaccao.2020.11.012 33842895 PMC8034586

[2] Kadowaki H , Akazawa H , Ishida J , Komuro I . Mechanisms and management of immune checkpoint inhibitor‐related cardiac adverse events. JMA J. 2021;4(2):91‐98. 10.31662/jmaj.2021-0001 33997442 PMC8118963

[3] Pardoll DM . The blockade of immune checkpoints in cancer immunotherapy. Nat Rev Cancer. 2012;12(4):252‐264. doi:10.1038/nrc3239 22437870 PMC4856023

[4] Brahmer JR , Lacchetti C , Thompson JA . Management of immune‐related adverse events in patients treated with immune checkpoint inhibitor therapy: American Society of Clinical Oncology clinical practice guideline summary. J Oncol Pract. 2018;14(4):247‐249. doi:10.1200/jop.18.00005 29517954

[5] Wang DY , Salem JE , Cohen JV , et al. Fatal toxic effects associated with immune checkpoint inhibitors. JAMA Oncol. 2018;4(12):1721‐1728. doi:10.1001/jamaoncol.2018.3923 30242316 PMC6440712

[6] Fazel M , Jedlowski PM . Severe myositis, myocarditis, and myasthenia gravis with elevated anti‐striated muscle antibody following single dose of ipilimumab‐nivolumab therapy in a patient with metastatic melanoma. Case Reports Immunol. 2019;2019:1‐3. doi:10.1155/2019/2539493 PMC6515062 31183226

[7] Jeyakumar N , Etchegaray M , Henry J , et al. The terrible triad of checkpoint inhibition: a case report of myasthenia gravis, myocarditis, and myositis induced by Cemiplimab in a patient with metastatic cutaneous squamous cell carcinoma. Case Reports Immunol. 2020;2020:1‐4. doi:10.1155/2020/5126717 PMC7355354 32695533

[8] Lipe DN , Galvis‐Carvajal E , Rajha E , Wechsler AH , Gaeta S . Immune checkpoint inhibitor‐associated myasthenia gravis, myositis, and myocarditis overlap syndrome. Am J Emerg Med. 2021;46:51‐55. doi:10.1016/j.ajem.2021.03.005 33721590

[9] Suzuki S , Ishikawa N , Konoeda F , et al. Nivolumab‐related myasthenia gravis with myositis and myocarditis in Japan. Neurology. 2017;89(11):1127‐1134. doi:10.1212/wnl.0000000000004359 28821685

[10] Pathak R , Katel A , Massarelli E , Villaflor VM , Sun V , Salgia R . Immune checkpoint inhibitor–induced myocarditis with myositis/myasthenia gravis overlap syndrome: a systematic review of cases. Oncologist. 2021;26(12):1052‐1061. doi:10.1002/onco.13931 34378270 PMC8649039

[11] Arora P , Talamo L , Dillon P , et al. Severe combined cardiac and neuromuscular toxicity from immune checkpoint blockade: an institutional case series. Cardiooncology. 2020;6:21. doi:10.1186/s40959-020-00076-6 32983574 PMC7513476

[12] Salem JE , Manouchehri A , Moey M , et al. Cardiovascular toxicities associated with immune checkpoint inhibitors: an observational, retrospective, pharmacovigilance study. Lancet Oncol. 2018;19(12):1579‐1589. doi:10.1016/S1470-2045(18)30608-9 30442497 PMC6287923

[13] Moslehi JJ , Salem J‐E , Sosman JA , Lebrun‐Vignes B , Johnson DB . Increased reporting of fatal immune checkpoint inhibitor‐associated myocarditis. Lancet. 2018;391(10124):933. doi:10.1016/s0140-6736(18)30533-6 PMC6668330 29536852

[14] Zhou Y , Hou Y , Hussain M , et al. Machine learning‐based risk assessment for cancer therapy‐related cardiac dysfunction in 4300 longitudinal oncology patients. J Am Heart Assoc. 2020;9(23):e019628. 33241727 10.1161/JAHA.120.019628 PMC7763760

[15] Mahmood SS , Fradley MG , Cohen JV , et al. Myocarditis in patients treated with immune checkpoint inhibitors. J Am Coll Cardiol. 2018;71:1755‐1764. 29567210 10.1016/j.jacc.2018.02.037 PMC6196725

[16] Palaskas N , Lopez‐Mattei J , Durand JB , Iliescu C , Deswal A . Immune checkpoint inhibitor myocarditis: pathophysiological characteristics, diagnosis, and treatment. J Am Heart Assoc. 2020;9(2):1‐12. doi:10.1161/jaha.119.013757 PMC7033840 31960755

[17] Bonaca MP , Olenchock BA , Salem JE , et al. Myocarditis in the setting of cancer therapeutics: proposed case definitions for emerging clinical syndromes in cardio‐oncology. Circulation 2019;140(2):80‐91. doi:10.1161/CIRCULATIONAHA.118.034497 31390169 PMC6779326

[18] Ferreira VM , Schulz‐Menger J , Holmvang G , et al. Cardiovascular magnetic resonance in nonischemic myocardial inflammation: expert recommendations. J Am Coll Cardiol. 2018;72(24):3158‐3176. doi:10.1016/j.jacc.2018.09.072 30545455

[19] Skouri HN , Dec GW , Friedrich MG , Cooper LT . Noninvasive imaging in myocarditis. J Am Coll Cardiol. 2006;48:2085‐2093. doi:10.1016/j.jacc.2006.08.017 17112998

[20] Johnson DB , Balko JM , Compton ML , et al. Fulminant myocarditis with combination immune checkpoint blockade. N Engl J Med. 2016;375(18):1749‐1755. doi:10.1056/NEJMoa1609214 27806233 PMC5247797

[21] Solimando AG , Crudele L , Leone P , et al. Immune checkpoint inhibitor‐related myositis: from biology to bedside. Int J Mol Sci. 2020;21(9):3054. doi:10.3390/ijms21093054 32357515 PMC7246673

[22] Okazaki T , Tanaka Y , Nishio R , et al. Autoantibodies against cardiac troponin I are responsible for dilated cardiomyopathy in PD‐1‐deficient mice. Nat Med. 2003;9:1477‐1483. doi:10.1038/nm955 14595408

[23] Michel L , Helfrich I , Hendgen‐Cotta UB , et al. Targeting early stages of cardiotoxicity from anti‐PD1 immune checkpoint inhibitor therapy. Eur Heart J. 2022;43(4):316‐329. doi:10.1093/eurheartj/ehab430 34389849

