# Supplementary material for: Immune checkpoint inhibitor induced myocarditis, myasthenia gravis, and myositis: A single‐center case series
Source: Cancer Med. 2022 Sep 21;12(3):2281–9. doi: 10.1002/cam4.5050 (PMC9939107; doi:10.1002/cam4.5050)
Supplement: Supplementary file 1 — Table S1 [file CAM4-12-2281-s001.docx]

**Table 1 Supplement.** Defining ICI-related myocarditis

| **Definite Myocarditis** | Diagnostic pathology  OR  Diagnostic CMRI + syndrome + EKG + Biomarkers  OR  Echo evidence + Syndrome + Biomarkers + EKG + negative LHC |
| --- | --- |
| **Probable Myocarditis** | Diagnostic CMRI  OR  Suggestive CMRI + syndrome + EKG or biomarkers  OR  Echo evidence + syndrome + EKG or biomarkers  OR  Syndrome + PET evidence + no alternate diagnosis |
| **Possible Myocarditis** | Suggestive CMRI  OR  Echo evidence + syndrome or EKG  OR  Biomarkers + syndrome  OR  EKG and no alternate diagnosis |

**Abbreviations:** PET = positron emission tomography, CMRI = cardiac magnetic resonance imaging, LHC = left heart catheterization. Definite, probable, and possible myocarditis as per proposed criteria by Bonaca et al.
